# Women’s Experiences of Promotion and Tenure in Academic Medicine and Potential Implications for Gender Disparities in Career Advancement: A Qualitative Analysis

**DOI:** 10.1001/jamanetworkopen.2021.25843

**Published:** 2021-09-20

**Authors:** Marie Murphy, Jacquelyn K. Callander, Daniel Dohan, Jennifer R. Grandis

**Affiliations:** 1 Department of Otolaryngology–Head and Neck Surgery, University of California, San Francisco; 2 Philip R. Lee Institute for Health Policy Studies, University of California, San Francisco

## Abstract

**Importance:**

Gender disparities in career advancement in academic medicine have persisted despite gender parity in medical school matriculation. Although numerous explanations for this gap exist, little is known about women’s experiences of promotion and tenure in academic medicine.

**Objective:**

To examine women’s experiences of promotion and tenure in academic medicine to uncover mechanisms associated with the gender disparity in career advancement.

**Design, Setting, and Participants:**

In this qualitative study, 52 in-depth, semistructured interviews with women academic medicine faculty members were conducted in 2019. The 52 participants were drawn from 16 medical schools across the US. Institutions were selected using a purposive sampling strategy to seek diversity of geography and ownership (private or public). Within institutions, purposive and snowball sampling were used to seek diversity with respect to respondents’ degree type (MD, PhD, and MD and PhD), age, and career stage. Interview transcripts were analyzed using qualitative thematic analysis. Data analysis was performed from March to December 2020.

**Main Outcomes and Measures:**

Themes and subthemes in participants’ experiences of promotion and tenure.

**Results:**

The 52 women in this study ranged in age from 34 to 82 years (mean [SD] age, 54.0 [10.7] years). Eighteen respondents (34.6%) held an MD, 4 (7.7%) held both an MD and PhD, and 30 (57.7%) held a PhD. Fourteen respondents (26.9%) were assistant professors at the time of the study, 8 (15.4%) were associate professors, and 30 (57.7%) were full professors. Four main themes within participants’ experiences of promotion and tenure that pertain to gender inequities were identified: ambiguous or inconsistent criteria for promotion or tenure; lack of standard processes for reviewing applications and making decisions; vulnerability to malicious behavior of senior faculty, department chairs, and division chiefs; and women seeing men have different experiences of advancement.

**Conclusions and Relevance:**

The respondents’ experiences of promotion and tenure suggest that promotion and tenure processes may be characterized by inconsistency and a lack of oversight, which have the potential to contribute to well-documented patterns of gender disparities in career advancement in academic medicine.

## Key Points

**Question:**

Are promotion and tenure processes associated with gender disparities in career advancement in academic medicine?

**Findings:**

In this qualitative study of 52 women at 16 medical schools across the US, participants experienced promotion and tenure processes to be poorly defined and inconsistently executed. They described a lack of recognition of or reward for their measurable accomplishments and observed their less-accomplished men colleagues advancing more quickly than they did.

**Meaning:**

These findings suggest that current mechanisms for promotion and tenure may create opportunities for unequal treatment and evaluation of candidates and may contribute to well-documented patterns of gender disparities in career advancement.

## Teaser

This qualitative study examines women’s experiences of promotion and tenure in academic medicine to uncover mechanisms associated with the gender disparity in career advancement.

## Introduction

For several decades, data have shown that women in academic medicine do not advance in their careers in parity with men.^1,2,3,4^ Women in academic medicine are promoted at lower rates than men and are less likely to hold tenured positions.^3,4^ Over a 35-year period, women physicians in academic medical centers were less likely than men to be promoted to the rank of associate or full professor, without any apparent narrowing in the gap throughout this time frame.^4^

Numerous explanations for gender disparities in advancement in careers in academic medicine have been proposed. The number of women applying to and matriculating into medical schools has steadily increased since the 1970s and is currently in parity with men, so many investigators have concluded that this gender gap likely cannot be attributed to a cohort effect or a weak pipeline.^2,3,5,6^ Much attention has been devoted to the likelihood that women will shoulder a disproportionate burden of family-related responsibilities within their households and the possibility that the most intensive phase of these responsibilities will coincide with and adversely affect their productivity during the most critical stage of their career development.^6,7,8,9^ Others note that implicit biases against women may impede their achievement of important markers of productivity, such as publication rates, upon which tenure and promotion decisions are predicated.^10^ Some researchers have found that women are less likely to be familiar with the criteria for promotion and tenure, potentially resulting from inadequate or insufficient mentoring.^11,12^ Others have reported that faculty on traditional tenure tracks are more likely to be promoted and that men are more likely than women to hold tenure-track positions.^13,14^ Implicit bias on the part of promotion and tenure committees may favor men, and criteria for tenure may be based on stereotypically masculine traits and behaviors, such as leadership.^1,10,15^ These diverse factors may occur in combination and have compounding effects.^10^

Despite the consensus that advancement of women in academic medicine has stalled, the copious literature dedicated to examining this phenomenon, and the various attempts medical schools have made to address gender disparities in promotion, we have limited information from women about their experiences of promotion and tenure.^8,16^ Data on how promotion and tenure policies and processes occur in everyday life have the potential to provide insights into why men continue to hold a persistent advantage over women in career advancement within academic medicine.

To partially address this gap in the literature, we used qualitative, in-depth interviews to examine women’s experiences of promotion and tenure, along with their observations of men’s career advancement in academic medicine. Examination of women’s experiences and perceptions of promotion and tenure is an essential component of a more comprehensive and nuanced understanding of how the processes of academic advancement may be associated with gender disparities in career advancement.

## Methods

### Study Design and Data Collection

This study was approved by a University of California, San Francisco, institutional review board, and all interviewees provided oral informed consent as required by the institutional review board. We report our study using the Consolidated Criteria for Reporting Qualitative Research (COREQ) reporting guidelines.

The data reported here are drawn from a larger qualitative study on gender inequities in academic medicine. The study team was led by a surgeon with extensive lived experience in academic medicine (J.R.G.) and 2 qualitative sociologists (D.D. and M.M.). One resident (J.K.C.) assisted with data organization and citation management. Our distinct perspectives informed the shared objective of understanding how women in academic medicine understand the association between gender and their professional experiences.^17^ The study adheres to social constructionist or constructivist approaches, which emphasize the importance of examining participants’ understandings of their own experiences when studying any social phenomena.^18,19^ We use a social constructionist conceptualization of gender, which considers the differences between women and men a product of social interactions and negotiations, rather than innate, biological differences.^20,21^

The senior author conducted in-depth, semistructured interviews with 52 women academic medicine faculty members at 16 institutions across the US. We recruited institutions using a purposive sampling strategy to seek diversity of geography and ownership (private and public). Within institutions, we used purposive and snowball sampling to seek diversity with respect to respondents’ degree type, age, and career stage. All interviews were conducted in 2019.

Participation in this research was voluntary, and interviewees were not compensated for their participation. We assured all participants of confidentiality with respect to both their identity and that of their institution. When interviewees were recruited upon the suggestion of another study participant, the interviewer did not disclose the source of the referral.

The development of the interview guide was informed by the senior author’s experience in academic medicine and the study team’s familiarity with the social science and academic medicine literature on gender equity and career advancement in academic medicine. Three pilot interviews were conducted to ensure that questions were sufficiently open-ended and attuned to the reality of participants’ experiences. Interview questions germane to this article are presented in Table 1. Interviews were conducted in a conversational, open-ended manner, as is standard practice for in-depth, semistructured interviews.^22,23^

**Table 1.** Interview Questions

Question category	Questions	Possible probes
Current position and professional history	What is your current position? How did you get to your current position?	Tell me about your… Experience of obtaining your first faculty position Experiences of going up for promotion/tenure High/low points in your career
Meritocracy in academic medicine	There is a widespread belief that academic medicine functions as a meritocracy. In your experience, how does the meritocracy function? Have you ever experienced or observed a situation in which the person who was most qualified for a position didn’t get the job?	Ask for details
Gender inequities in academic medicine	Have you experienced challenges or difficulties in your career that you think might be associated with or attributable to your gender? Have you ever seen or heard of a colleague experiencing challenges or difficulties in academic medicine that could have been related to their gender? Have you seen or heard of a colleague experiencing advantages in academic medicine that could have been related to their gender? Why do you think we continue to have a “leaky pipeline” in academic medicine? (What are your impressions of the reasons why women leave academic medicine at higher rates than men?)	Ask for details

Two interviews were conducted via Zoom, and the rest were conducted in person. Interviews lasted approximately 60 minutes and were digitally recorded and professionally transcribed verbatim.

### Statistical Analysis

The interview transcripts were entered into ATLAS.ti qualitative data analysis and research software version 8 (Scientific Software Development GmbH) for storage and qualitative data analysis. We (M.M., J.R.G., and D.D.) established a set of a priori codes, based on the senior author’s lived experiences in academic medicine and our familiarity with literature on gender inequities and academic medicine. The code “promotion/tenure” was developed in this manner, whereas subcodes such as “seeing men advance more quickly” emerged inductively on the basis of reading and discussion of salient themes within the first 10 interview transcripts. This article is based on analysis of portions of the interview transcripts coded “promotion/tenure,” which included participants’ discussions of their own experiences of promotion and tenure and their observations of others’ experiences of promotion and tenure.

After the initial set of codes was developed, we read 10 additional interviews, coded them individually, and then convened to discuss our interpretations of the data and applications of codes. We addressed differences in our analyses and then revised the codes’ inclusion and exclusion criteria accordingly. We then coded another 10 interviews using the refined codebook, and upon comparison of our individual efforts, found that intercoder agreement, as defined by Campbell et al,^24^ had been achieved. Subsequently, 2 members of the study team (M.M. and J.R.G.) applied a subset of codes to all interviews, following the standard practices in qualitative data analysis of keeping reflective memos and regularly discussing the application of codes.^25^ Data analysis was performed from March to December 2020.

## Results

Fifty-six women were invited to participate in the study, 52 agreed, and 4 declined. Those who declined to participate cited scheduling conflicts. The 52 women who participated had a mean (SD) age of 54.0 (10.7) years. Table 2 displays information about the study participants. Eighteen of these women (34.6%) held an MD degree, 4 (7.7%) held both an MD and a PhD, and 30 (57.7%) held a PhD. Fourteen of the women (26.9%) were assistant professors, 8 (15.4%) were associate professors, and 30 (57.7%) were full professors at the time they were interviewed. Twenty-three (44.2%) held leadership positions, and 15 (28.8%) held endowed chairs. We identified 4 dominant themes and subthemes within our interviewees’ experiences and observations of promotion and tenure, which are listed in Table 3 with illustrative quotes.

**Table 2.** Study Participants’ Age, Degree Type, and Career Status

Characteristic	Participants, No. (%) (N = 52)
Age, mean (SD) [range], y	54.0 (10.7) [34.0-82.0]
Degree	
MD	18 (34.6)
MD and PhD	4 (7.7)
PhD	30 (57.7)
Professor rank	
Assistant	14 (26.9)
Associate	8 (15.4)
Full	30 (57.7)
Leadership position	23 (44.2)
Endowed chair	15 (28.8)

**Table 3.** Themes, Subthemes, and Illustrative Quotes From Women Faculty in Academic Medicine

Subthemes	Illustrative quotes
Theme 1: Ambiguous or inconsistent criteria for promotion or tenure	
Criteria for promotion not clearly defined or communicated	“I was told that I couldn’t be a professor because I needed to travel more internationally. My chief put me up for promotion, but they said, ‘Sorry, you need more international invitations to demonstrate your international reputation.’ Which is just not going to happen.” Participant 12
Moving goalpost	“When you actually ask for tenure, it all comes down to money. So I [obtained grant funding], and came back to them and said, ‘Now I’m coming with money. I have research. I teach. I formed a track on a master’s program. I do everything. Can you tell me, am I ready now?’ They wouldn’t say yes, but they wouldn’t say no. They said, ‘Well, you’ve got to wait.’ So I started talking to people. My attitude was, ‘Screw this, I am going to make this happen.’ I went to my chair, and he said, ‘I can’t help you. I want to promote you, but it’s not in my hands.’” Participant 71
Lack of recognition for measurable accomplishments	“I contacted one of the individuals on the Promotion and Tenure Committee to solicit feedback on my CV and she felt it might be appropriate for me to go up, so I emailed my chair and told him I wanted to begin a discussion about my eligibility. But then this turned into a discussion about the strength of my publication record, and that it might warrant pulling me off the tenure track. This was not what I expected. I had just gotten my first R01, and they’re talking about taking me off the tenure track? Where is that coming from?” Participant 102
Denial of promotion even when accomplishments were recognized	“I was up for promotion to associate this year, and I submitted everything, and the next thing I know is that my promotion was blocked. I’m unclear on why it didn’t go through, and I’m still trying to get feedback. The main thing I was able to get from my division chief was that it was too early for me. But in the letter that denied my promotion, it said my accomplishments have exceeded those at my level.” Participant 7
	“I’d been there for about 9 years when a tenure-track job opened up. I applied for the job and they had me give a seminar, and I ran around and interviewed with everybody. And then they hired a guy who was far less qualified than I was. There was one woman [on the committee] who told me, ‘You know, they didn’t even discuss you, even though you were by far the most qualified applicant for that job.’ Later, I found out that they felt I was captive. I was sitting outside of the chairman’s office, and he was talking to someone, and he said, ‘Well, [she and her husband] won’t leave because the school system is so good here.’ That was the common thought process within the administration: if a husband and a wife were both there, and had kids in the school system, they were captive.” Participant 85
Theme 2: Lack of standard processes for reviewing applications and making decisions	
Concerns about progression of promotion or tenure process	“I’ve had to go and ask about promotion, set up my own meetings, pound on doors to [initiate the promotion process]. And then, when we finally submitted the application, I had to consistently pound on the door, and I definitely had the sense that I was perceived as being aggressive and impatient for advocating for myself. My expectation was that if I was going through the process, somebody would be keeping an eye on it and giving me regular updates. Instead, months and months would go by and nothing would happen. I would write and say, ‘Are we moving forward?’ I’d get a response from the chief saying, ‘Oh, I was out sick. I didn’t get to write the letter.’ I had the experience that I was being perceived as being aggressive for asking for normal things.” Participant 15
Power of chairs to delay promotion process	“I went up for promotion to associate, and [my chair] sat on my promotion for a year.” Participant 61
Theme 3: Vulnerability to malicious behavior of senior faculty, department chairs, and division chiefs	
Denial of tenure without forewarning	“Two of my [men] faculty mentors denied me tenure, instead of telling me they didn’t think I was ready or telling me they had concerns. It was horrible; nervous-breakdown horrible. But I fought, and it was challenging, but I got tenure.” Participant 49
Attempted removal by chairman	“It was clear my chairman didn’t like me…and he’s supposed to be in charge of my future. At one point he told me that he was going to put me up for early tenure—I don’t even think it was my third year. I was two years into a six-year tenure track, and I said, ‘No.’ Six months later he comes back and tells me the committee in our department says I have to go up and I don’t have a choice. At the time, I didn’t realize what was going on. What happened was, when I started my job, the letter with my salary offer had not been approved by the right people. They came back to my chair and told him he had to pay the difference in my salary from his departmental slush fund. He was trying to remove me.” Participant 86
Theme 4: Women seeing men have different experiences of advancement	
Seeing men with lesser accomplishments advance more quickly	“I continuously saw male colleagues who were much less accomplished than me being promoted. They would be my colleagues, and then they would be my supervisors, and when I looked at it on paper, there was no question that I had more grants. I had more papers. I was better known for what I was doing.” Participant 108
Being told it was too early to advance	“When I wanted to go up for tenure and promotion to associate, I was at year five on a seven-year clock, and I went to my chief and he told me it was too early for me. He said, ‘No, when the time is right, we tell you, you don’t come and tell us.’ But then people who had come up behind me went up for promotions. And they were men. And the chief put them up. I took my CV to the vice dean for research and asked her opinion, and she said, ‘This is ridiculous, you should have gone up.’” Participant 25
Explicit messages that tenure decisions were related to gender	“[When I went up for tenure] I just had a *Nature* paper published, I do this associate directorship job, I started the cancer bio program. I had done all this stuff and I felt like my science was good, too. But when I finally got any insight into how the process was going, [the committee] said, ‘She will get tenure because she’s a woman, but we don’t want her to get tenure just because she’s a woman.’ So I got three male colleagues’ CVs and showed what their CVs looked like when they were put up for tenure, and they finally put me up and I got it. But it was a fight.” Participant 8

Abbreviation: CV, curriculum vitae.

### Theme 1: Ambiguous or Inconsistent Criteria for Promotion and Tenure

#### Criteria for Promotion Not Clearly Communicated or Defined

Many women we interviewed told us that criteria for promotion or tenure were not made clear, and that there was always the potential to be surprised by unknown requirements. One woman (participant 12) learned about the requirement of international travel for promotion to full professor 8 months before being interviewed for this study, after having served on the faculty of her institution for more than 20 years. Her experience demonstrated the potential for promotion requirements to be poorly defined, in addition to being insufficiently communicated: her robust research collaboration with an investigator in Europe and their coauthored publications could have been considered evidence that her work had international impact.

#### Moving Goalpost

Another woman (participant 71) explained that even when she endeavored to find out exactly what she needed to do to be awarded tenure, the goalpost moved as soon as she accomplished the stated requirements. In addition, the people who had the authority to grant her tenure provided vague answers when she asked about the sufficiency of her qualifications. This woman “fought hard” for tenure, and eventually received it, but described the fight as “two years of nonsense.”

#### Lack of Recognition for Measurable Accomplishments

Other women we interviewed were surprised by a lack of recognition for accomplishments that are widely considered significant within academic medicine. One woman (participant 102), who was a basic scientist in a clinical department, described how the merit of her National Institutes of Health R01 funding was not recognized by her chair: he did not know what an R01 was and did not recognize that it was a huge achievement when she went up for tenure.

#### Denial of Promotion Even When Accomplishments Were Recognized

Even when women’s professional accomplishments were explicitly recognized, they were not necessarily considered sufficient for promotion. One woman (participant 7) was denied promotion despite the committee’s recognition that her accomplishments exceeded those of others at her level. Another woman (participant 85) was not promoted to a tenure-track position, despite the committee’s recognition that she was the most qualified applicant for the job. This participant reported overhearing her chairman saying that she and her husband (who worked at the same institution) would never leave, because they had children in school and the school system was so good, indicating that factors outside of her professional accomplishments might have factored into her promotion decision.

### Theme 2: Lack of Standard Processes for Reviewing Applications and Making Decisions

#### Concerns About the Progression of the Tenure or Promotion Process

Women found that going up for promotion and/or tenure was no guarantee that their application would be reviewed promptly or that anyone with authority over the process would actively manage its progress. One woman (participant 15) described having to “consistently pound on the door” to ensure her promotion was progressing through the review process. She got the message that she was perceived as being aggressive for advocating for herself and checking on the status of her promotion, but even so, the process moved slowly.

#### Power of Chairs to Delay Promotion Process

Another woman (participant 61) told us that her chair, who was a man, “sat on” her promotion for a year. The reason, as far as she could discern, was that the chair was new to his role.

### Theme 3: Vulnerability to the Malicious Behavior of Senior Faculty, Department Chairs, and Division Chiefs

#### Denial of Tenure Without Forewarning

Although many women in our study believed that people who were in charge of their promotion or tenure did not assist them as much as they could have, several women described experiences with senior faculty who had power over their advancement which they interpreted as deliberate attempts to undermine them. For instance, 1 woman (participant 49) was denied tenure by 2 faculty members who could have let her know they had concerns about her qualifications before she went up.

#### Attempted Removal by Chairman

Another woman (participant 86) discovered that her chairman wanted to eliminate her position. She sought help from the provost, who was ostensibly charged with protecting faculty. The provost (who was a woman) told her that if she made trouble, things could get worse for her, and indeed, the provost handled this interviewee’s information in a manner that allowed her request for assistance to get back to her chairman, which only made her situation more fraught.

### Theme 4: Seeing Men Have Different Experiences

#### Seeing Men With Lesser Accomplishments Advance More Quickly

When the women we interviewed compared their career advancement with their observations of their men colleagues’ careers, they sometimes observed that men with achievements comparable to theirs advanced more quickly than they did. For instance, participant 108 told us that although she had more grants and publications than her men colleagues and was better known for what she was doing than they were, the men advanced more quickly than she did.

#### Being Told It Was Too Early to Advance

Women also described being told it was too early for them to go up for promotion or tenure. Meanwhile, men in their departments with similar or lesser qualifications were put up.

#### Explicit Messages That Tenure Decisions Were Related to Gender

A few women in our study received explicit messages that their promotion or tenure decisions were directly related to their gender, such as participant 8, who was told that the tenure committee did not want her to get tenure “just because she’s a woman.” In response, she showed the committee the curriculum vitae of 3 men colleagues who had been put up for tenure to demonstrate her worthiness, and this measure convinced them to award her tenure.

## Discussion

Gender disparities in promotion and tenure outcomes in academic medicine have been studied extensively and documented; this qualitative study adds to the literature by using in-depth interviews to examine women faculty members’ experiences with the processes of promotion and tenure. Our interviews with 52 women at 16 institutions across the US surfaced themes that add new considerations to the substantial literature on gender disparities in promotion and tenure in academic medicine.

Prior studies^10,26^ on gender disparities in career advancement have noted that different types of work tend to be valued differently in academic medicine, and that women are more likely to do more of the work that does not have as much prestige, such as teaching and service, whereas men are more likely to do more high-prestige work, such as obtaining grant funding or publishing in high-profile journals. However, our interviewees described a different problem: a lack of recognition for accomplishments that are generally considered prestigious within academic medicine, such as obtaining National Institutes of Health R01 funding or a publication in a high-impact scientific journal such as *Nature*. Furthermore, even when a committee explicitly recognized the strength of a woman’s professional accomplishments, they did not necessarily reward them with advancement. These findings suggest a possible absence of clearly defined and communicated criteria for promotion or tenure—or the possibility that if clearly defined criteria for promotion and tenure exist, they may not be consistently applied.

In addition to believing their own measurable accomplishments were not always recognized or rewarded appropriately, women observed men having different experiences of advancement than they did. Women saw their objectively less-accomplished men colleagues put up for promotion and being promoted earlier than they were. Whether or not their impressions of the fairness of their men colleagues’ advancement were factually accurate is not the main point. These impressions further support the possibility that criteria for promotion and tenure may be ill-defined, poorly communicated, or applied inconsistently, and that under such circumstances, there is potential for both inequitable treatment and the perception of inequitable treatment to occur. Both outcomes are important. Previous research^6^ has found that women sometimes feel undervalued by their institutions; our findings are consistent with these results and suggest that women may sometimes have legitimate reasons for such a sentiment.

Although previous studies^11,12,16^ have documented that some participants in academic medicine believe that women fail to advance their careers in parity with men because they do not understand what is required of them as faculty members and what is required to achieve promotion and tenure, our study illustrates an entirely different set of possibilities. When the women in our study did not know exactly what they needed to do in order to advance their careers, they made significant efforts to find out and persisted even when they received vague answers from their superiors. However, meeting the ostensible criteria for advancement was no guarantee of success. Women in our study found the criteria could be changed, or their measurable accomplishments might not be recognized, or not considered sufficient for promotion even if others with similar qualifications had been promoted.

### Limitations

This study has limitations that should be considered. The criteria for promotion and tenure are different for different types of faculty, on different types of career tracks, and differ from institution to institution. Although our study provides rich data on participants’ experiences of promotion and tenure, we did not attempt to examine how degree type or career track affected their experiences or to identify patterns in respondents’ experiences within or between institutions, nor did we attempt to ascertain the presence or content of promotion and tenure policies at our respondents’ institutions to complement our examination of our participants’ experiences of promotion and tenure. In addition, our study did not attend to diversities of identity within the gender category of women. It also did not examine the significance of additional dimensions of diversity, such as sexual orientation, race/ethnicity, and disability, which may have important implications for gender disparities in career advancement in academic medicine.

## Conclusions

Our findings indicate the utility of further research on promotion and tenure processes and policies in academic medicine. Future studies might usefully examine the generalizability of our findings to larger populations or specific subpopulations. Studying the prevalence and content of formalized promotion and tenure policies and their association with promotion and tenure practices could also usefully enhance our understanding of how gender disparities are produced—or potentially mitigated—within promotion and tenure processes. Previous interventions that have been implemented or proposed to address gender disparities in career advancement, such as better mentoring for women, leadership development courses for women, family-friendly policies, and unconscious bias training for committees, may well have value,^16^ but our research suggests that making tenure and promotion practices more transparent and subject to systematic oversight could also have broad impacts, both for improving individuals’ experiences of promotion and tenure and for addressing gender disparities in career advancement.
